# Child wasting is a severe public health problem in the predominantly rural population of Ethiopia: A community based cross–sectional study

**DOI:** 10.1186/s13690-017-0194-8

**Published:** 2017-06-12

**Authors:** Amare Tariku, Gashaw Andargie Bikis, Haile Woldie, Molla Mesele Wassie, Abebaw Gebeyehu Worku

**Affiliations:** 1 Department of Human Nutrition, Institute of Public Health, College of Medicine and Health Sciences, University of Gondar, Gondar, Ethiopia; 2 Department of Health Service Management and Health Economics, Institute of Public Health, College of Medicine and Health Sciences, University of Gondar, Gondar, Ethiopia; 3 Department of Reproductive and Child Health, Institute of Public Health, College of Medicine and Health Sciences, University of Gondar, Gondar, Ethiopia

**Keywords:** Wasting, Children under five, Poor dietary diversity, Ethiopia

## Abstract

**Background:**

In Ethiopia, child wasting has remained a public health problem for a decade’s, suggesting the need to further monitoring of the problem. Hence, this study aimed at assessing the prevalence of wasting and associated factors among children aged 6–59 months at Dabat District, northwest Ethiopia.

**Methods:**

A Community based cross-sectional study was undertaken from May to June, 2015, in Dabat District, northwest Ethiopia. A total of 1184 children aged under five years and their mothers/caretakers were included in the study. An interviewer-administered, pre-tested, and structured questionnaire was used to collect data. Standardized anthropometric body measurements were employed to assess the height and weight of the participants. Anthropometric body measurements were analyzed by the WHO Anthro Plus software version 1.0.4. Wasting was defined as having a weight–for–height of Z–score lower than two standard deviations (WHZ < −2 SD) compared to the WHO reference population of the same age and sex group. In the binary logistic regression, both bivariate and multivariate analyses were done to list out factors associated with wasting. All variables with P–values of < 0.2 in the bivariate analysis were earmarked for the multivariate analysis. Both Crude Odds Ratio (COR) and Adjusted Odds Ratio (AOR) at 95% Confidence Interval (CI) were computed to determine the strength of association. In the multivariate analysis, variables at *P*–values of < 0.05 were identified as determinants of wasting.

**Results:**

The overall prevalence of wasting was 18.2%; 10.3% and 7.9% of the children were moderately and severely wasted, respectively. Poor dietary diversity [AOR = 2.08, 95% CI: 1.53, 4.46], late initiation of breastfeeding [AOR = 1.43, 95% CI: 1.04, 1.95], no postnatal vitamin-A supplementation [AOR = 1.55, 95% CI: 1.04, 2.30], and maternal occupational status [AOR = 2.31, 95% CI: 1.56, 3.42] were independently associated with wasting in the study area.

**Conclusion:**

Wasting is a severe public health problem in Dabat District. Therefore, there is a need to strengthen the implementation of optimal breastfeeding practice and dietary diversity. In addition, improving the coverage of mothers^’^ postnatal vitamin-A supplementation is essential to address the burden of child wasting.

## Background

Undernutrition is the most devastating problem affecting the majority of the world’s children [1]. That poor nutritional status during childhood has long–lasting scarring consequences [2]. Undernutrition diminishes the working capacity of an individual during adulthood [3], and it silently destroys the future socio-economic development of nations [2]. Ultimately, it causes the vicious cycle of intergenerational undernutrition [4].

Underweight, stunting, and wasting are the three main indicators used to define undernutrition [5], which is having a Z–score lower than two standard deviations as compared to the reference population of the same age and sex [6]. Low weight-for-height (WHZ ≤ −2 SD) is an indicator of wasting, which is generally associated with recent illness and child failure to gain weight [7].

Worldwide, 52 million children under five years of age are wasted [8] and most of the global burden of wasting (acute undernutrition) is found in developing countries [7, 9, 10]. Likewise, the magnitude of the problem is substantial and persistent in Sub–Saharan Africa (SSA) [11]. Different studies in Ethiopia reported a high prevalence of wasting among children aged under–five years [12–16]. The 2014 Ethiopia Demographic and Health Survey report showed that, about 9% of children are wasted [17].

Causes of child undernutrition depend on complex interactions of various factors, such as socio–demographic, environmental, cultural, and political [18–20]. Many studies have been conducted to investigate the magnitude and predictors of wasting in Ethiopia [21, 22] and other parts of the world [23–27]. Findings revealed that household economic status [10, 17, 28, 29], mothers residence [29, 30], educational status [31], occupation [32] and nutritional status [7], child morbidity status [8, 31–33], sex, age [32, 34, 35], age of initiation into complementary feeding [14, 15], and birth interval [36] are associated with wasting.

Since the last two decades, Ethiopia has been thriving to improve the level of malnutrition in different segments of the population [37]. On the other hand, the magnitude and complications of acute undernutrition remain a public health problem in the country [17]. Wasting is among the leading nutritional problems, causing morbidity and mortality in children under five years of age [15]. Evidence based health and nutrition findings have a crucial role in improving the level of wasting and mortality reduction in children [38]. However, studies identifying factors associated with wasting among children aged under five years in Northwest Ethiopia are scarce. Thus, this study aimed at assessing factors associated with wasting among children aged under five years at Dabat District, Northwest Ethiopia. The findings from the study will provide evidences to programme managers and policymakers for designing and implementing appropriate interventions to mitigate the level of malnutrition and wasting in particular, among children aged under five years in Northwest Ethiopia.

## Methods

### Study setting

A community-based cross-sectional study was conducted from May to June, 2015 at Dabat Health and Demographic Surveillance System (HDSS) site, located in Dabat District, northwest Ethiopia. The district has an estimated population of 145,458, living in 26 rural and 4 urban kebeles *(smallest administration units Ethiopia)*. The inhabitants mainly depend on subsistence farming. The HDSS covers 67,385 people, living in thirteen kebeles (four urban and nine rural) selected by considering the different ecological zones (high land, middle land, and low land). The Dabat HDSS site has been operational since November, 1996.

### Sampling procedure and study population

Initially, the study aimed at assessing the nutritional status and feeding practice of children aged 6–59 months at the Dabat HDSS site. Of the total thirteen kebeles in the HDSS, eight were selected by using the lottery method. Accordingly, all mothers with children aged 6–59 months and lived in the selected kebeles for at least six months were included in the study. For households with multiple children, only one was selected using the lottery method. Sample size was determined using Epi-info version 3.7 by considering the following assumptions; the prevalence of wasting in Ethiopia as 9% [17], 95% level of confidence, 5% margin of error, 10% non-response rate, and a design effect of 2. Thus, the minimum sample size of 844 was obtained. However, the 1184 eligible children found in the original survey were included in order to improve the power of the investigation.

### Data collection instrument and procedures

Interviewer administered, pre-tested, and structured questionnaire was used to collect data. The questionnaire was designed with three major factors in mind. The first part was on socio-demographic and economic related characteristics of the child and family. The second part involved feeding patterns, morbidity status, and health care utilization related characteristics of the child, while the third section focused on hygiene and sanitation related characteristics of the household/family. To maintain the consistency of the data, the questionnaire was first translated from English to Amharic *(the native language of the study area)* and was retranslated to English by English language and public health experts. Fourteen data collectors and three field supervisors (working at Dabat DHSS) were recruited for the study. The recruits were given two days training on the objective of the study, ethical concerns, and data collection techniques. The tool was piloted on 5% of the total sample out of the study area. During the pre–test, the applicability of the data collection procedures was evaluated. The questionnaire was checked for completeness and clarity by the respective supervisors on a regular basis.

The anthropometric measurement was done according to the standardized procedures stipulated by the Food and Nutrition Technical Assistance (FANTA) Anthropometric Indicators Measurement Guide [39]. The stature of the child was measured using the seca vertical height scale (German, Serial No. 0123) with the child standing upright in the middle of the board. The child’s head, shoulders, buttocks, knees, and heels touched the vertical board. The length of a child (aged 6–23 months) was measured using a horizontal wooden length board in a recumbent position and read to the nearest 0.1 cm.

Child weight was measured to the nearest 0.1 kg by the seca beam balance (German, Serial No. 5755086138219) with a graduation of 0.1 kg and a measuring range of up to 25 kg. Weight was taken in light clothing and no shoes. Instrument calibration was done before weighing each child. Furthermore, the weighing scale was checked daily against the standard weight for accuracy. Each child’s height and weight measurements were repeated, and the mean value was calculated and recorded on the copies. Anthropometric-related data were transferred to the WHO Anthro Plus software version 1.0.4. The weight-for-height Z-score (WHZ) was calculated using the WHO Multicenter Growth Reference Standard. A child whose weight-for-height z score was less than −2 standard deviations (WFH < − 2 SD) from the reference population was defined as wasted [40].

Dietary diversity scores (DDSs) were estimated by using a 24-h recall method. Individual DDSs were meant to reflect the micronutrient adequacy of the diet. The scores were validated for several age/sex groups as proxy measures for macro and micronutrient adequacy of the diet. Furthermore, DDSs have positively correlated with the micronutrient density to complementary foods for infants and young children [41]. The scores were also used to monitor progress or target interventions at population levels [42]. The recall period of 24 h is the mostly chosen technique of many studies as it is less subject to recall bias, less cumbersome for respondents, and conforms to the recall period [43]. Moreover, the analysis of dietary diversity data based on a 24-h recall period is easier than other longer dietary assessment recall periods [44]. The mothers were requested to list out what food groups were consumed by their children in the previous 24–hours prior to the date of survey. The DDS of four is considered as the minimum acceptable dietary diversity; accordingly, a child with a DDS of less than four was classified as having poor dietary diversity; otherwise, it was considered to have good dietary diversity [45].

### Data analysis

Data were entered into EPI-info version 3.5.3 and exported to the Statistical Package for Social Sciences (SPSS) version 20 for analysis. Descriptive statistics, including frequencies and proportions, were used to summarize the variables. Maternal employment status was categorized into three groups, i.e. housewife, farmer, and others (e.g. unemployed, student, and servants). Binary logistic regression was used to investigate factors associated with wasting. All those variables with a p-value of < 0.2 in the bi-variable analysis were entered into the multivariable regression analysis. The Adjusted Odds Ratio (AOR) with a 95% confidence interval was estimated to assess the strength of association, and a *p*-value of < 0.05 was used to declare statistical significance in the multivariable analysis.

Using the principal component analysis (PCA), household wealth index was computed by considering properties like, selected household assets and size of agricultural land. In the PCA, the Eigen value of greater than one, KMO distribution, and a communality value of greater than 0.5 were used to select variables for the final model. In the final model, the selected variables were summed and ranked into lowest, middle, and highest.

## Results

The mean (Standard Deviation, ±SD) age of the children was 27.7 (±14.0) months. About 15.2% of the children were aged 6–11 months. About 30.1% and 32.9% of the mothers and fathers of the children attended formal education, respectively. More than fifty percent (52.2) of the households had five and less family members. A good proportion (69.4%) of the households accessed their food from their own farms (Table 1).

**Table 1:** Socio–demographic and economic characteristics of study participant at Dabat District, northwest Ethiopia, 2015

Variables	Frequency	Percent
Child sex		
Male	598	50.5
Female	586	49.5
Child age (in months)		
6–11	180	15.2
12–36	693	58.5
37-59	311	26.3
Maternal age (in years)		
15–34	678	57.3
35–50	506	42.7
Marital status of the mother		
Currently unmarried	131	11.1
Currently married	1053	88.9
Religion		
Orthodox	1122	94.8
Others^a^	62	5.2
Mother’s educational status		
No formal education	822	69.6
Formal education	359	30.4
Mother’s employment status		
Housewife	683	57.7
Farmer	314	26.5
Others^b^	187	15.8
Father’s educational status		
No formal education	794	67.1
Formal education	390	32.9
Health care access of the household		
Good	1046	88.3
Poor	138	11.7
Household size		
≤ 5	618	52.2
> 5	566	47.8
Sources of food for household consumption		
Own production	822	69.4
Purchasing	320	27.0
Others^c^	42	3.6
Household wealth status		
Poor	447	37.8
Medium	355	30.0
Rich	382	32.2

^a^Muslim and protestant

^b^unemployed, student, and servants

^c^donation and gift from relatives

Almost all (99.3%) of the children were ever breastfed, about 52.3% initiated breastfeeding within one hour of delivery, while two-thirds (62.9%) of them remained exclusively breastfed for six months. About 37.2% and 17.9% of the participants had fever and diarrheal episodes, respectively. More than three-quarters (79.4%) of the children received vitamin-A supplementation, and one-third (35%) took a deworming tablet. The majority (94.1%) of the children had poor dietary diversity. Only a quarter (24.7%) and half (45.3%) of mothers received postnatal vitamin-A and prenatal iron-folate supplementation, respectively (Table 2).

**Table 2:** Feeding pattern, health care, and morbidity related characteristics of children aged under five years at Dabat District, northwest Ethiopia, 2015

Variables	Frequency	Percent
Ever breastfeeding		
Yes	1176	99.3
No	8	0.7
Initiation breastfeeding		
early initiation	619	52.3
late initiation	565	47.7
Colostrum feeding		
Yes	615	51.9
No	569	48.1
Pre-lacteal feeding		
Yes	341	28.8
No	843	71.2
Exclusive breastfeeding		
Yes	745	62.9
No	439	37.1
Initiation of complementary feeding		
Timely (6 – 8 Months)	685	57.9
Early (<6 Months)	144	12.2
Lately (≥9 Months)	355	30.0
History of bottle feeding		
Yes	58	4.9
No	1126	95.1
Deworming status of the child		
Yes	414	35.0
No	770	65.0
Child vitamin–A supplementation in the past six month		
Yes	940	79.4
No	244	20.6
History of fever in the past two weeks		
Yes	440	37.2
No	744	62.8
History of diarrheal morbidity in the past two weeks		
Yes	212	17.9
No	972	82.1
Dietary diversity score		
< 4 food groups	1114	94.1
≥ 4 food groups	70	5.9
Maternal feeding status during pregnancy		
Less than before	488	41.2
As usual	674	56.9
Greater than before	22	1.9
Postnatal Vitamin–A supplementation		
Yes	292	24.7
No	892	75.3
Prenatal Iron supplementation		
Yes	536	45.3
No	648	54.7

About 36.1% of households used protected sources of water, and 28.7% had latrines. Nearly three-fourths of the mothers washed their hands after toilet, whereas almost all (95.9%) washed their hands before feeding their children (Table 3).

**Table 3:** Hygiene and sanitation related characteristics of household's at Dabat District, northwest Ethiopia, 2015

Variables	Frequency	Percent
Source of drinking water for the household		
Protected	428	36.1
Unprotected	756	63.9
Time to fetch water		
≥ 30 min	866	73.1
> 30 min	318	26.9
Household drinking water treatment practice		
Not at all	1092	92.2
Always	65	5.5
Sometimes	27	2.3
Latrine availability		
Yes	340	28.7
No	844	71.3
Hand washing practice after toilet		
Not at all	132	11.1
Sometimes	204	17.2
Always	848	71.6
Hand washing practice before feeding the child		
Not at all	10	0.8
Sometimes	38	3.2
Always	1136	95.9
Household waste disposal practice		
Appropriate	144	12.2
In appropriate	1040	87.8
Child faces disposal practice		
Appropriate	243	20.5
In appropriate	941	79.5

The overall prevalence of wasting was 18.2% [95% CI: 15.8, 20.3], of which 10.3% and 7.9% were moderately and severely wasted, respectively.

### Determinants of wasting

The result of the multivariate logistic regression analysis showed that time for initiation into breastfeeding, maternal postnatal vitamin-A supplementation, and occupation status were identified independent determinants of wasting at 95% confidence intervals. Accordingly, children with poor dietary diversity were more likely to have wasting [AOR = 2.08, 95% CI: 1.53, 4.46] compared to those who had good dietary diversity. Similarly, increased odds of wasting were noted among children whose mothers initiated breastfeeding after one hour of delivery [AOR = 1.43, 95% CI: 1.04, 1.95] and received no postnatal vitamin-A supplementation [AOR = 1.55, 95% CI: 1.04, 2.30]. Furthermore, the odds of wasting were higher among mother engaged in other work categories [AOR = 2.31, 95% CI: 1.56, 3.42] compared to housewife mothers (Table 4).

**Table 4:** Factors associated with wasting among children aged under five years at Dabat District, northwest Ethiopia, 2015

Variables	Wasting			
	Yes	No	Crude Odds Ratio	Adjusted Odds Ratio
Initiation of breastfeeding				
Early initiation	96	523	1	1
Late initiation	119	446	1.45 (1.08,1.96)	1.43 (1.04,1.95)^*^
Vitamin-A supplementation in the past one year				
Yes	178	762	1	1
No	37	207	0.77(0.56, 1.13)	0.79 (0.53, 1.18)
Complementary feeding initiation				
Timely	108	577	1	1
Early	27	117	1.23(0.77, 1.96)	1.12 (0.71, 1.86)
Late	80	275	1. 55 (1.13, 2.15)	1.27 (0.91, 1.79)
Exclusive breastfeeding				
Yes	124	621	1	1
No	91	348	1.31 (0.97, 1.77)	0.94 (0.59, 1.49)
Dietary Diversity Score				
Poor (<4 food groups)	207	907	1.77 (0.83, 375)	2.08 (1.53, 4.46)^*^
Good (≤4 food groups)	8	62	1	1
Main source of food				
Own production	686	136	1	1
Purchasing	245	75	1.54(1.12, 2.12)	1.19 (0.81, 1.72)
Other	38	4	0.53(0.19, 1.51)	0.49 (0.17, 1.44)
Wealth status				
Poor	85	362	1.03 (0.73, 1.46)	0.96 (0.67, 1.48)
Medium	59	296	0.87(0.59, 1.27)	1.08 (0.72, 1.62)
Rich	71	311	1	1
Maternal vitamin-A supplementation				
Yes	38	254	1	1
No	177	715	1.66 (1.13, 2.42)	1.55 (1.04, 2.30)^*^
Pregnancy feeding practice				
As usual	72	416	1	1
Less than the pre-pregnancy	141	533	1.53(1.12, 2.09)	1.66 (0.98, 2.28)
Greater than the pre-pregnancy	2	20		0.59 (0.14, 2.64)
Marital status				
Currently unmarried	38	93	2.02 (1.34, 3.05)	0.76 (0.47, 1.25)
Currently married	177	876	1	1
Mother’s employment				
Housewife	101	582	1	1
Farmer	62	252	1.42 (1.00, 2.01)	1.27 (0.89, 1.82)
Other	52	135	2.22 (1.51, 3.26)	2.31 (1.56, 3.42)^*^
Bottle feeding				
Yes	13	45	1.32 (0.70, 2.50)	1.26 (0.65, 2.43)
No	202	924		1

*Significant at a *P–*Value of < 0.05

## Discussion

In this study, the prevalence of wasting was 18.2%, suggesting a severe public health problem according to the Nutrition Landscape Information System (NLIS) cut-off values [46]. The finding was consistent with the recent EDHS report [17], and other previous local studies, like East and West Gojjam (17.1-18.6%) [47], Somali Region (17.5%) [48], Bulehora District (13.4%) [49], and East Harargie (11.2%) [50]. A similarly high prevalence of wasting was reported from the three regions of Allahabad (10.6%) [51], Karnataka (16%) [52], and Bhubaneswar, India (23.3%) [53]. The high burden of wasting in these study settings may be explained in terms of the similarity that the majority of the participants share by living in rural areas. Obviously, poor child feeding practices and a high prevalence of food insecurity are the commonly reported determinants of wasting [47–50, 53], as documented in the rural communities of Ethiopia [51, 54].

However, the result was higher than what was reported from other developing countries, such as Ghana (4.7%) [55], Kenya (2.1%) [56], and Iran (0.7%) [57]. The disparities could be attributed to the better socio-economic status of the population in the latter study areas. In fact, children in better-off families have improved opportunity to get nutritious and diversified food [57]. On the other hand, compared to what was done in Kenya and Iran, this study involved a larger number of participants which might have contribution to the higher prevalence of wasting.

The result of the multivariate analysis revealed that late initiation of breastfeeding, poor dietary diversity, no postnatal vitamin–A supplementation, and maternal employment status were significantly and independently associated with wasting. Accordingly, the odds of wasting were higher among children with late initiation into breastfeeding. This is similar to various reports in Ethiopia and elsewhere [1, 8, 15, 24], confirming the fact that early initiation into breastfeeding helps the newborn to get the nutritional and protective benefits of the colostrum [58]. In addition, it promotes suckling, successful establishment, and maintenance of BF throughout infancy [59]. Despite its benefits, many mothers delay the initiation of breastfeeding, that is, only 43% of newborns in developing countries are put to the breast within one hour of birth. Establishing good breastfeeding practices in the first days is critical to the health and nutritional status of the infants. Also, it is one of the proven nutrition intervention for saving lives [60].

This study indicated that the likelihood of wasting was higher among children with poor DDSs. Poor dietary diversity, a proxy indicator of poor diet quality and nutrient intake of children [61], negatively influences the nutritional status of children [62, 63]. Providing nutrient-rich foods in sufficient quantity and quality starting at a child’s age of six months is one of the long-term and effective strategies to reduce child undernutrition [64, 65]. However, mother/caretaker IYCF knowledge is still low and a major problem in developing countries [66, 67]. As a result, children living in most developing countries are introduced directly to regular household diets made of cereal or starchy root crops [68] which are poor in micronutrient density [69].

The likelihood of developing wasting was high among children whose mothers were in other employment categories. Other studies also reported that children of unemployed mothers were at increased risk of developing wasting [5, 29, 30, 32]. This is further evidenced by the fact that unemployment of mothers is among the strong indicators of socio-economic resources of the household [70], sustaining a strong negative influence on household earnings and the level of food security [10]. It is clear that food insecure households have a limited capacity to afford and eat well-diversified diets [71]. This results in the family, particularly children eating foods poor in quality and quantity [28]. Furthermore, the powerlessness of women in the household is commonly documented to be the fate of unemployed mothers [72].

Similarly, the odds of wasting increased among children whose mothers received no postnatal vitamin-A supplementation. The concentration of vitamin-A and other micronutrients (iodine, thiamin, riboflavin and pyridoxine) in breast milk is dependent on the level of mother’s body store and dietary intake [73]. Hence, poor vitamin-A status of mothers at the time of pregnancy and postnatal period, determines the amount of vitamin-A in the breast milk [74, 75], for breast feeding a child. Child vitamin-A deficiency is correlated with a high risk of developing infectious diseases, including diarrhea and respiratory tract infections [76, 77], which are the commonly reported predictors of wasting [48–50]. Furthermore, postnatal visit also creates opportunity for mothers to get health and nutrition counseling, which is crucial to address their socio-cultural misconceptions on IYCF practice [78–80].

The study used a large sample size to show the burden of wasting in a well defined population . However, some of the limitations of the study should be considered. Firstly, the co-morbidity status of the child was conveyed only by information given by mothers/caregivers. This might be subjected to bias, as it may depend on the mothers/caretakers’ level of knowledge of the illness status of the child. Secondly, measurement of child feeding practice again relied on memory, so there was a possibility of recall bias.

## Conclusion

The magnitude of wasting is high in Dabat District which suggest a severe public health concern in northwest Ethiopia. Wasting was associated with initiation of breastfeeding, dietary diversity, maternal postnatal vitamin-A supplementation, and employment status. Therefore, there is a need to strengthen implementation of the current IYCF strategy to improve the dietary diversity and breastfeeding practice. In addition, improving the coverage of mother's postnatal vitamin-A supplementation is essential to address childhood wasting.

## Abbreviations

AOR: Adjusted odds ratio
CI: Confidence interval
COR: Crude odds ratio
DDS: Dietary diversity score
FANTA: Food and nutrition technical assistance
HDSS: Health and demographic surveillance system
PCA: Principal component analysis
SD: Standard deviation
WHO: World health organization
WHZ: Weight for height Z-score
